# Professor Mahmood Bhutta on disrupting unhealthy supply chains and promoting environmental sustainability in health care

**DOI:** 10.1017/ash.2023.533

**Published:** 2024-01-11

**Authors:** Mahmood F. Bhutta

**Affiliations:** 1 Department of Clinical and Experimental Medicine, Brighton and Sussex Medical School, Brighton, East Sussex, UK; 2 Department of ENT, University Hospitals Sussex, Brighton, East Sussex, UK

## Can you tell our readers about yourself, your training, and how you became interested in labor issues and sustainability?

I am an ENT surgeon working within the National Health System in the UK specializing in ear surgery, but I’ve also had a long-standing interest in health systems, and half my work is now academic, on the research and publication side of things. I first became inspired to do this work when I was training to be a surgeon. Interestingly, my parents are from a town in Northern Pakistan called Sialkot which produces 70% of the world’s surgical instruments. When I was there on my honeymoon visiting family, one of my cousins asked if I wanted to visit a surgical manufacturing plant. So, I went and what I saw shocked me. There were people working in utterly unacceptable conditions, children as young as 7 years old, making these instruments that would ultimately be used by the National Health Service. That was business, as usual, for them, but for me, this represented a very unhealthy way for us to obtain our supplies. I then contacted the British Medical Association, and we set up a group to examine worker rights pertaining to healthcare products. Now I work with my University, Brighton and Sussex Medical School, where we established the Sustainable Healthcare Group, to examine harmful labor practices and the environmental harms from our supply chains.^1^

## How did you start this work, what sort of funding or support was required and how did it come together?

It was a bit of good fortune—I had been working on supply chains for some time, and one of my junior doctors wanted to pursue a research project on sustainability. Eventually, we created a PhD to start carbon foot printing the products we use in health care. When I started, there were people saying, “why are you investigating that? Is that even important?” This was a few years ago, and now, of course, I am glad to say that the sustainability agenda is much more of a mainstream concept. Now we’ve developed some strong evidence, which means that we can speak with authority and direct change. So, in fact, we’ve just launched a national report called “Green Surgery.”^2^ It’s 100 pages long with 500 references and is an evidence-based set of recommendations, endorsed by all the surgical colleges in the UK, as well as the Australian and Canadian College. In this report, we address use of surgical equipment and make other recommendations in terms of care pathways.

## Please share with our readers examples of abuses and poor practices you witnessed as pertains to medical and surgical equipment and other healthcare practices. What is at risk if we do not “clean up our act” in health care?

An example I’ve already mentioned is adults and children working excessive hours receiving extremely poor wages to manufacture healthcare products. In an article I wrote some years ago on the harms caused by our healthcare supply chains,^3^ I found major issues with gloves. I learned that 70% of the world’s gloves are manufactured in Malaysia, where there is endemic use of forced migrant labor from countries such as Bangladesh, Nepal, and Indonesia. Our survey of 1500 workers showed that many paid 2000 and up to 5000 US dollars in illegal recruitment fees to come to Malaysia to work.^4^ They will work 13-hour days, typically without a single day off for the first 3 months, and often their entire existence centers on working in the factory, to be transported by factory bus to some factory accommodation. Their passports were confiscated, and they had no freedom. In the worst cases, I’ve seen workers that have been imprisoned in the factory because of a dispute with management. I’ve also seen pictures of workers beaten with sticks and wounds from these injuries that I don’t share in my presentations because they’re too unpleasant. Furthermore, we know some of our masks are unfortunately manufactured using forced labor. For instance, in the northwest of China, where there has effectively been genocide of Uighur populations, and we know that some masks for health care have been manufactured using State-sponsored forced Uighur labor. In the eastern part of China, there were findings that some healthcare gowns were made where between 70% and 100% of the salary of migrant North Korean workers was sent to the North Korean State.

## Can you expand on how these unethical labor practices drive unsustainability in health care?

I call these “unhealthy supply chains.” We have become accustomed to throwing things away because of a misunderstanding of the risk of reusing equipment in health care. We developed this notion based on illnesses like HIV, mad cow disease, and now COVID, where we initially thought everything was potentially infectious. Rather than dealing with processes to downgrade the risk of infection, we start to just throw things away, which seems an easier solution. This means, of course, that we’re throwing away the embedded carbon and causing other environmental harms through the extraction of resources from our planet. We’re also throwing away money and our ability to help and respect the workers making this equipment. When we prioritize quantity, price becomes the primary driver. When the price of raw materials for manufacturing is the same all over the world, then profit stems from how little you’re prepared to invest in the people that make these products for us. Labor rights abuses through global supply chains seem to be rampant in health care.

## Our readership consists primarily of professionals who work in Infection Prevention and Antimicrobial Stewardship. What daily practices in these fields contribute to the climate crisis and what are the things we can do right now to improve?

We need the infection prevention community to be part of the solution. Those I speak to at a senior level recognize that we have become too risk-averse and that the reaction to risk is to throw things away. So, we need the infection prevention community to be the leaders of change, advance their own science, and spread their knowledge and understanding to other groups in the hospital. For example, in the UK we know that two-thirds of our glove use is inappropriate. People use gloves all the time rather than recognizing that clean hands are the best way to prevent infection. We can reduce our glove use by educating people that they should wash their hands rather than use gloves for routine patient care. We have also come to rely on a culture of disposable textiles because of historic concerns about materials such as cotton, which have problems with water penetration. But modern materials in high-income settings do not have those problems; we have access to textiles that are impervious to water; therefore, rather than perpetuating an economy incentivizing disposable products, we need to move to reusable drapes and gowns, because this practice is better for the planet, with no risk to patients. Another thing that comes to mind in my daily practice are disposable plastic textiles to hold instruments in the operating theater, which could easily be replaced by a reusable metal container. Why do we have these disposable plastic textiles? Unfortunately, we are incentivized to use things and throw them away rather than paying for equipment to be used, sterilized, and reused.^4^

## Why do you think so many people in health care are unaware of these unhealthy supply chains and practices?

I think many people in health care have not considered the effects of throwing everything away on our planet and on human health. Where do you think your cheap clothes or cheap agricultural products come from? It’s the same in health care, and I suppose that’s what I’ve been trying to show. Every healthcare institution in high-income settings must have an infection prevention program but people appointed to run these programs may not be empowered to enact brave or even sensible policies. We also have economies with a linear model of consumption; we keep selling things, and now it seems difficult to reverse course and say, “no, we don’t want to buy more things.”

## In your travels around the Europe and the world giving lectures, what are some sustainability initiatives that have impressed you that you think can be implemented universally?

In the UK, my group has been campaigning for moving towards reusable textiles and gowns, and that should become the norm in surgery. Recent data shows that in the UK last year, the proportion of reusable drapes and gowns has increased by 17%, so, we’re having some effect. In the central region of Denmark, they have committed to a policy where they will reuse all medical equipment wherever possible, and they’re building the infrastructure to enable that. Sweden and Norway have collaborated to deal with the labor rights issues in glove manufacturing and have been a potent force for change there. The United States has taken a more forceful approach to labor rights issues and glove manufacturing, where five glove suppliers were banned from supplying to the United States because of illegal working conditions, which has had a measurable impact on the Malaysian industry, where the loss of income forced manufacturers to improve worker conditions. While this approach remains controversial, I don’t doubt that it has had the biggest effect on worker rights in the glove industry in the last few years.

## What challenges have you faced?

I believe that most people in health care really want their work and everything they do to be sustainable in terms of not harming our planet or others. But there are some personal challenges I have faced, particularly from companies who are supplying only single-use items. I’ve been mocked by people who say that you cannot change these things. I’ve been harassed at a conference by a major supplier of single-use endoscopes but thankfully that was dealt with by the conference organizers. I’ve been censored and disinvited from speaking at a sustainability conference for not agreeing to avoid mention of reusable drapes and gowns due to pressure from a supplier of single-use drapes and gowns. I’ve also been threatened with legal action by manufacturers of gloves for highlighting issues with labor rights in their factories, which were later proven to be true. These are some of the challenges I face, but I won’t be shut up.

## So, where do you find the courage to continue?

What I try to speak is the truth, but I’m happy to be challenged. I derive courage from having seen what happens to these workers with my own eyes. It is wrong to stand by or agree to be silenced. I’m also enthused by the number of people at a senior level, who support everything I’m doing, and so in that, I have that safety amongst friends in positions of influence and power.

## How do you engage young people who are just starting in medicine or nursing careers to adapt more sustainable practices?

It’s an easy win. The younger generation are very much on board with this as they see it as important and part of their future and the future of their patients. They can envision a future where we don’t throw everything away. When you’re starting out, it can be difficult because you don’t have the power, but I encourage them to be ambassadors for change and ambassadors of common sense. One practical thing that young doctors and nurses can do is look at all the medications that patients are on and discontinue the ones that are not needed. Medications are another major area of concern; 20% of the carbon footprint of the National Health Service in the UK is due to medications. And, they can adopt the sensible use of gloves.

## What are one or two major sustainability initiatives from policymakers that you’d like to see happen?

I would like to see to reuse of products in health care to become the norm, and if we’re going to throw anything away, there must be an absolute justification. I would like to see transparency around decisions to make something disposable rather than reusable, and we need the infrastructure to support that across the world. We also need more transparency about where our products come from and the realities of workers making these products. We need real worker empowerment and unionization. Their voices need to be heard so we understand the truth of how workers are being abused in our supply chains. We cannot supply health care at the cost of other humans.

## So far, everything you’ve discussed pertains to daily healthcare practices. What can we do in non-patient-care domains to really have influence?

Obviously, our responsibility as healthcare workers also extends to our personal lives and our daily decisions. We can cycle or walk to work if feasible, we can consume much less meat, which is more carbon-friendly and healthier, and we can take reusable mugs for our coffee or tea. In the UK, I’m glad to say it’s very difficult to purchase coffee or tea that is made under bad working conditions, and the same applies to most of our agricultural products. But we should be asking this of every product we consume and the garments we wear. We need to start understanding the realities of the choices we make every day.

## What accomplishments are you most proud of to date? Where do you see yourself in 5–10 years? What do you hope to have achieved in terms of health care and sustainability initiatives across the world?

The papers provide the evidence to move the agenda forward and the policies outline what we need to do, but if it all just sits on a shelf, looking pretty, that doesn’t help anyone. So, we need real-world change. The fact that we’ve increased the number of reusable textiles by 17% in the UK is heartening to hear. There are tangible benefits to that sort of change. Many people I’ve spoken with have said, “I’ve stopped wearing gloves for many of these scenarios, and I’ve stopped using this piece of equipment.” In terms of worker rights, I’m glad to have contributed to a consortium that led to 150 million US dollars being repaid to workers in Malaysia. So, I know things are improving for them, but there’s still a lot of work to do. Where do I see myself in 5 or 10 years? Probably still harking on about this because changing global supply chains is not easy! But on a positive note, after working on these issues for nearly 20 years now, I’m glad to say that this conversation is now mainstream, and we can now move the conversation from “why?” to “how?”

## Finally, which books, podcasts, or articles would you recommend to our readers to better understand the issues, what is at stake, and how to start to fix these problems in health care?

I became interested in climate change when I was a teenager. I read a book by James Lovelock, who was a British independent scientist and environmentalist who developed a concept called “Gaia” which described the interconnectedness of everything living on earth.^5^ He was ridiculed at the time but ended up working for NASA, which led to work showing that there was a hole in the ozone layer. His ideas are now mainstream; the whole planet is one living being. As a teenager, I also read Naomi Klein’s book “No Logo,” which describes in detail the problems with the garment and agricultural industries contributing to worker abuses.^6^ These books cultivated for me a desire and a moral obligation, to delve deeper into these issues, as pertained to my own life and career.
